# Mediation of PKM2-dependent glycolytic and non-glycolytic pathways by ENO2 in head and neck cancer development

**DOI:** 10.1186/s13046-022-02574-0

**Published:** 2023-01-02

**Authors:** Lixia Gao, Fan Yang, Dianyong Tang, Zhigang Xu, Yan Tang, Donglin Yang, Deping Sun, Zhongzhu Chen, Yong Teng

**Affiliations:** 1 National & Local Joint Engineering Research Center of Targeted and Innovative Therapeutics, Chongqing Key Laboratory of Kinase Modulators as Innovative Medicine, College of Pharmacy & International Academy of Targeted Therapeutics and Innovation, Chongqing University of Arts and Sciences, Chongqing, 402160 China; 2 Chongqing Academy of Chinese Materia Medica, Chongqing, 400065 China; 3 Department of Hematology and Medical Oncology, Winship Cancer Institute, Emory University School of Medicine, 201 Dowman Dr, Atlanta, GA 30322 USA; 4 University-Town Hospital of Chongqing Medical University, Chongqing Medical University, Chongqing, 401331 China

**Keywords:** ENO2, PKM2, HNSCC, Glucose metabolism, Cell cycle, AP-III-a4

## Abstract

**Background:**

Enolase 2 (ENO2) is a crucial glycolytic enzyme in cancer metabolic process and acts as a “moonlighting” protein to play various functions in diverse cellular processes unrelated to glycolysis. ENO2 is highly expressed in head and neck squamous cell carcinoma (HNSCC) tissues relative to normal tissues; however, its impact and underlying regulatory mechanisms in HNSCC malignancy remain unclear.

**Methods:**

Molecular alterations were examined by bioinformatics, qRT-PCR, western blotting, immunofluorescence, immunohistochemistry, immunoprecipitation, and ChIP-PCR assays. Metabolic changes were assessed by intracellular levels of ATP and glucose. Animal study was used to evaluate the therapeutic efficacy of the ENO inhibitor.

**Results:**

ENO2 is required for HNSCC cell proliferation and glycolysis, which, surprisingly, is partially achieved by controlling PKM2 protein stability and its nuclear translocation. Mechanistically, loss of ENO2 expression promotes PKM2 protein degradation via the ubiquitin-proteasome pathway and prevents the switch of cytoplasmic PKM2 to the nucleus by inactivating AKT signaling, leading to a blockade in PKM2-mediated glycolytic flux and CCND1-associated cell cycle progression. In addition, treatment with the ENO inhibitor AP-III-a4 significantly induces HNSCC remission in a preclinical mouse model.

**Conclusion:**

Our work elucidates the signaling basis underlying ENO2-dependent HNSCC development, providing evidence to establish a novel ENO2-targeted therapy for treating HNSCC.

**Supplementary Information:**

The online version contains supplementary material available at 10.1186/s13046-022-02574-0.

## Background

Head and neck squamous cell carcinoma (HNSCC) develops from the mucosal epithelium in the oral cavity, pharynx and larynx and are on the rise, with annual rates increasing from 4% to as much as 10% in recent years [1, 2]. Alcohol, tobacco, and human papillomavirus (HPV) infection are well known causative factors for HNSCC. Clinical and genomic meta-analysis of multicohort cancer gene expression profile has revealed that HPV(+) and HPV(-) HNSCC subtypes differ with respect to the molecular mechanisms underlying their oncogenic processes [3]. Despite advances in clinical management over the past decade, the overall 5-year survival rate in patients with advanced HNSCC remains unsatisfactory. Besides surgery, radiotherapy and chemotherapy, only three targeted agents (cetuximab, nivolumab and pembrolizumab) are approved by the FDA to combat HNSCC, but the majority of patients cannot benefit from them [4–6]. These clinical challenges prompt efforts to identify the key molecular determinants involved in HNSCC malignancy and develop novel treatment options to better treat HNSCC patients.

Cancer cells maintain distinctive energy metabolism networks to support cell survival, growth, progression, and metastasis under harsh conditions [7]. Glycolysis is the first step in the breakdown of glucose to extract energy for cellular metabolism. In glycolysis, the glucose uptake by cancer cells is higher than in healthy cells, and lactic acid is produced from pyruvate that reduces the pH of the tumor [8]. Moreover, the high (18^F^) fluoro-2-deoxy-D-glucose (FDG) uptake by the HNSCC cells potentially correlates with their glucose uptake [9, 10]. Our previous study further demonstrated that most HNSCC cell lines are “glucose addicted” or “glutamine addicted” [11] and fast generation of energy during glycolysis promotes cell proliferation in rapidly growing HNSCC cells, suggesting the priority of glycolytic utilization specifically to HNSCC cells. Therefore, targeting the metabolic requirements of HNSCC may guide antitumor treatment strategies.

Enolase 2 (ENO2), also known as human neuron-specific enolase (NSE) or γ-enolase, is a rate-limiting enzyme in glycolysis promoting the conversion of 2-phosphoglycerate (2-PG) to phosphoenolpyruvate (PEP) [12, 13]. ENO2 mainly exists in neurons and neuroendocrine tissues, which is a long-chain acidic dimer protein containing 433 amino acids that includes two enolate isoenzymes *γγ* and *αγ* [14]. ENO2 is a well-established tumor biomarker in various types of cancers, including pancreatic ductal adenocarcinoma (PDAC), prostate cancer, metastatic neuroblastoma, small-cell lung cancer (SCLC), and the microvascular invasion status of liver cancer [12, 15]. Recently, overexpression of ENO2 has been found to be associated with increased cell proliferation and glycolysis enrichment in papillary renal cell carcinoma [10]. The same tendency was also seen in bone marrow mononuclear cells in hematological tumors [11]. Nevertheless, there have not been many mechanistic investigations into the role of ENO2 in HNSCC to date. There is some evidence from metabolomic studies indicating that aerobic glycolysis is the major route of carbon metabolism in HNSCC [16]. Thus, defining the role and mechanisms of ENO2 in HNSCC development may help identify novel counter strategies to combat this deadly disease.

Pyruvate kinase M2 (PKM2) is a key rate-limiting enzyme in glycolysis that catalyzes the final step in glycolysis, which is associated with tumor metabolism and growth [17]. In cancer cells, PKM2 expression is upregulated, whereas the expression of tissue-specific PKM1 derived from a single PKM gene by alternative splicing of a primary mRNA transcript declines [18]. It has become clear from past research that PKM2 is involved in both glycolytic and non-glycolytic pathways and is instrumental in the malignancy of tumor cells through its metabolic and nonmetabolic functions [19, 20]. Here, we demonstrate, for the first time, that ENO2 drives HNSCC cell proliferation and glucose metabolism via the dual role of PKM2 by regulating its protein stability and nuclear translocation. Based on this study, we suggest that ENO2 acts as an oncogenic driver in HNSCC development and targeting it using the ENO inhibitor AP-III-a4 represents a potential therapeutic approach for treating this type of cancer.

## Materials and methods

### Cell lines and human primary specimens

Human-derived HNSCC cell lines Cal27, Cal33, SCC15 and SCC25 were purchased from ATCC (Rockville, MD) and UM-1, HSC3 cells were purchased from Japanese Collection of Research Bioresources (JCRB) Cell Bank (Japn). All cell lines were maintained in culture no more than 10 passages according to the supplier’s instructions. Human telomerase-immortalized tonsillar keratinocytes hTERT HAK Clone 41 were a gift from Dr. A. Klingelhutz and Dr. J. Lee (University of Iowa, Iowa City, IA) and cultured in KSFM with 0.2 ng/ml EGF and 30 μg/ml bovine pituitary extract [21]. Human primary HNSCC specimens of paraffin-embedded tissue blocks were obtained from the University-Town Hospital of Chongqing Medical University, China. Specimens were collected and processed in compliance with protocols approved by the Institutional Review Board of Chongqing Medical University. Human subjects provided informed consent in the course of this research.

### Inhibitors, antibodies, and standard assays

ENO inhibitor AP-III-a4 (ENOblock), PKM2 inhibitor compound 3k, AKT inhibitor AZD5363 and MEK inhibitor U0126 were obtained from SelleckChem (Houston, TX). Cycloheximide (CHX) and MG132 were purchased from Sigma-Aldrich (St Louis, MO). Nuclear Extraction Kit was purchased from Abcam (Cambridge, United Kingdom). Glycolysis antibody sampler kit, including PKM2, glyceraldehyde-3-phosphate dehydrogenase (GAPDH), hexokinase I (HK I), hexokinase II (HK II), lactate dehydrogenase A (LDHA), phosphofructokinase, platelet (PFKP) and pyruvate dehydrogenase (PDH) antibodies, were purchased from Cell Signaling Technology (Beverly, MA). ERK1/2, p-ERK1/2, AKT, p-AKT, P70S6K, p-P70S6K, cyclin D1 (CCND1), cyclin B, cyclin E, CDK2, CDK4, CDK6 and ENO1 antibodies were purchased from Cell Signaling Technology. Ubiquitin, HA and β-actin antibodies were purchased from NovusBio (Centennial, CO). Cell viability was determined by MTT assay and crystal violet (0.5%, v/v) staining. Standard cell culture, cell proliferation, quantitative RT-PCR (qRT-PCR), western blotting, plasmid transfection and lentiviral infection were carried out as we described previously [22, 23].

### Constructs and gene modifications

The pLVX-puro lentiviral vectors containing an shRNA against ENO2 were purchased from Invitrogen (Waltham, MA). The full-length HA-tagged ENO2 and PKM2 were cloned into the pDONR-221 lentiviral expression vector (Invitrogen). After transfection and puromycin selection, the positive clones of ENO2 knockdown (shENO2), ENO2 overexpression (ENO2 O/E) and PKM2 overexpression (PKM2 O/E) were screened by western blotting with antibodies against ENO2 and PKM2.

### Confocal immunofluorescence (IF)

Cell fixation was performed using 4% paraformaldehyde in PBS. After permeabilization with 0.25% Triton X-100, cells were treated with blocking buffer for 30 min and incubated with PKM2 antibody (1:200) overnight at 4°C, followed by incubation with the secondary antibody at room temperature for 1 h. The coverslips were mounted onto glass slides and counterstained with DAPI (Sigma-Aldrich) and images were captured using OLYMPUS BX61 LASER Scanning Confocal Microscope.

### Soft agar colony formation

Soft agar colony formation assays were carried out using a two-layer soft agar system as we previously described [23]. Briefly, approximately 1×10^4^ cells suspended in 1.5 ml of top agar (0.3% agar in 2× DMEM complete cell culture medium) were overlaid onto a layer of 1.5 ml of bottom agar (0.6%) for 28 days, and the colonies were photographed and counted under an inverted phase-contrast microscopy.

### Cell cycle analysis

Cell cycle progression was assessed by flow cytometry. Briefly, the gene modified cells and their corresponding control cells were cultured in 6-well plates for 48 h. Cells were then collected, washed twice with cold PBS, fixed in 70% ethanol at 4°C for 24 h and stained with PI/RNase for 30 min. The cell cycle analysis was performed on the Accuri C6 (BD Biosciences, Mountain View, CA, USA) and the data were analyzed by Flow Jo software (FACSCalibur).

### Measurement of glucose uptake and intracellular ATP concentration

To determine the glucose levels, 5×10^4^ gene modified cells and their corresponding control cells were cultured in a 96-well plate overnight. The culture medium was removed, and cells were washed with PBS. Cells were then incubated with fresh-prepared 500 μM 2-deoxyglucose (2-DG, 50 μL/well) for 20 min at room temperature. The uptake process was stopped and neutralized, and luciferase activities were measured by Glucose Uptake-Glo Assay Kit (Promega, Madison, WI). Intracellular ATP concentration was measured by ATP Assay Kit (Colorimetric) (Abcam, Waltham, MA) following the manufacturer’s instructions. All values were normalized by the corresponding total protein level.

### Chromatin immunoprecipitation coupled with qPCR (ChIP-qPCR) assay

ChIP assays were performed using a ChIP assay kit (Millipore Sigma, Burlington, MA) as described previously [16, 17]. The eluates were immunoprecipitated with a control IgG or anti-PKM2 antibody. Each immunoprecipitated DNA sample was quantified by qPCR using the primers (F: 5’-TCTACACCGACAACTCCATCCG-3’; R: 5’-TCTGGCATTTTGGAGAGGAAGTG-3’) that were designed to amplify a proximal promoter region containing putative PKM2 binding sites on the CCND1 gene promoter. All samples were run in triplicate, and results were averaged after normalization to the input.

### CHX chase assay and protein immunoprecipitation (IP)

For CHX chase assay, indicated HNSCC cells were treated with 100 μg/ml of CHX for 9 hours maximally. Western blotting was then performed to determine the half-life of the PKM2 protein. For IP assay, the lysed cells were incubated with anti-HA antibody, followed by addition of protein A/G Sepharose® beads (Amersham Biosciences, South San Francisco, CA) overnight at 4 °C on a rotating platform, followed by western blotting with indicated antibodies.

### Animal study and drug administration

Six-week-old immunodeficient nude mice were purchased from the Experimental Animal Center of Chongqing Medical University (Chongqing, China). All animal experiments were approved by the Institutional Animal Care and Use Committee (IACUC) of Chongqing University of Arts and Sciences. To generate a xenograft tumor model in nude mice, 1×10^6^ UM-1 cells were suspended in 100 μl of PBS/Matrigel (3:1) and injected subcutaneously. When the tumors reached ~100 mm^3^, the tumor-bearing mice were randomly assigned into two different groups to receive vehicle (*n=*6) and AP-III-a4 (*n=*8) treatment. AP-III-a4 was given by intraperitoneal injection every other day for 28 days at a dose of 20 mg/kg body weight. Tumor volume measurement was performed by digital caliper according to the formula V = length × width^2^ × 1/2. At the experimental endpoint, primary tumor xenografts and major organs (including the heart, liver, spleen, lung, kidney, and intestine) from the mice were excised and processed for histopathological analysis.

### Immunohistochemistry (IHC) assays

IHC assays were performed as we previously described [11, 21]. Briefly, slides were deparaffinized, rehydrated, incubated in 3% H_2_O_2_ to block endogenous peroxidase activity. After antigen retrieval processed by boiling in sodium citrate for 30 min, slides were blocked by using 10% goat serum for 15 min, followed by incubation with specific primary antibodies (ENO2, 1:200; ENO1, 1:500;  Ki67, 1:100; p-AKT, 1:500; p-ERK, 1:500; and PKM2, 1:100) at 4 °C overnight. Then slides were washed 3 times, incubated with the second antibody at room temperature for 30 min, washed 3 times and incubated with diaminobenizidine (DAB) for 3 min. Finally, the nuclei were counterstained with Mayer’s hematoxylin. The final immunoreactivity score of ENO2 in human specimens was examined by three investigators who were blind to pathological information by using the German semi-quantitative scoring method as we previously described [24]. IHC staining intensity of targeted proteins in xenograft tumors was quantified based on five areas of each sample from xenograft sections using Image pro-Plus6.0 software (Media Cybernetics, Silver Springs, MD) and presented as integrated optical density (IOD).

### Bioinformatics and statistical analysis

Data on the expression of ENO1, ENO2, ENO3 and PKM2 genes for 33 cancer types and adjacent non-carcinoma tissues were extracted from TCGA (https://tcga.xenahubs.net) and used to generate an expression matrix. Raw data was summarized by means, standard deviations (SD), and graphical summaries and transformed if necessary to achieve normality. Data from the in vitro experiments are presented as means ± SD from three independent experiments. Statistical analyses were performed by unpaired Student’s *t* test for two group comparisons and one-way analysis of variance (ANOVA) for multi-group comparisons at a significance level of *p*<0.05.

## Results

### Correlation between ENO2 expression level and HNSCC development

**Fig. 1:**
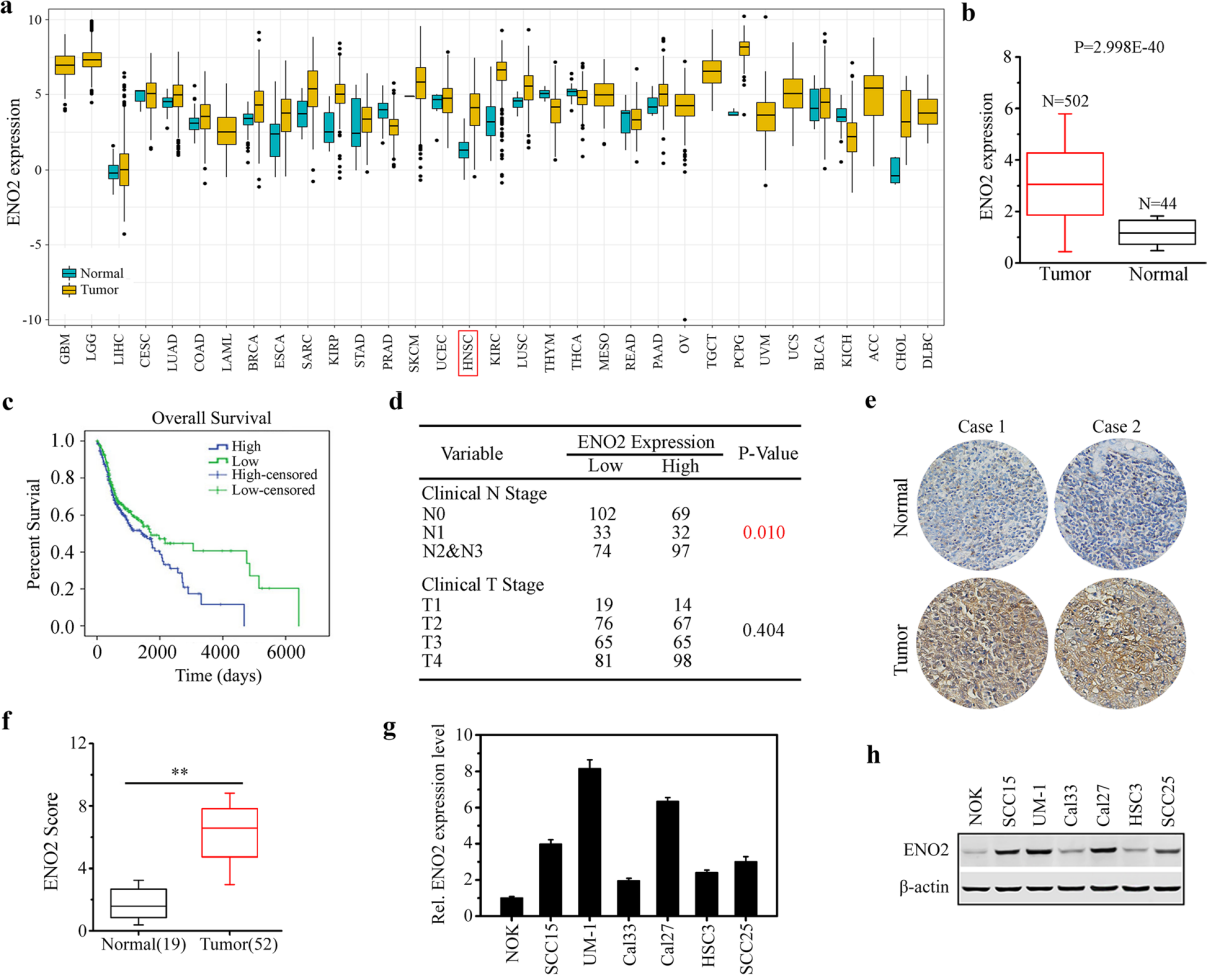
Correlation between ENO2 expression level and HNSCC development. **a** Analysis of ENO2 expression in normal and tumor tissues using TCGA cohort with 33 cancer types. **b** The expression levels of ENO2 in head and neck normal and tumor tissues using GEO dataset. **c** Kaplan-Meier survival analysis of the correlation between the expression levels of ENO2 and survival in HNSCC patients. **d** Chi-square test analysis of the correlation between the expression levels of ENO2 and clinical stage of HNSCC patients. **e**, **f** ENO2 expression levels in human primary HNSCC samples (*n=*52) and normal tissues (*n=*19) determined by IHC. Representative IHC result and quantitative data are shown in e and f, respectively. **g**, **h** Transcriptional (**g**) and protein levels (**h**) of ENO2 in different HNSCC cell lines determined by qRT-PCR and western blotting, respectively. **p<0.01

To explore whether the ENO2 gene contributes to the occurrence and development of cancer, we used bioinformatics techniques to analyze ENO2 expression in normal and tumor tissues using TCGA cohort with 33 cancer types. Pan-cancer analysis showed significant differential expression of ENO2 in 12 TCGA tumors, including HNSCC (Fig. 1a). Analysis of an independent GEO database confirmed significantly increased levels of ENO2 expression in HNSCC tissues compared with normal samples (Fig. 1b). Kaplan-Meier survival analysis revealed that the high expression of ENO2 was strongly correlated with the poor overall survival in HNSCC patients (Fig. 1c). Further Chi-square test analysis showed that ENO2 expression levels were significantly associated with clinical N grade, but not clinical T grade, suggesting the role of ENO2 in HNSCC development (Fig. 1d). We then collected primary human HNSCC samples and normal tissues and performed IHC with anti-ENO2 antibody. IHC data confirmed that the expression level of ENO2 in HNSCC tissues was significantly higher than in normal tissues regardless of anatomic site (Fig. 1e-f). ENO1 was slightly increased in HNSCC tissues relative to the normal counterparts, but this increase did not achieve statistical significance (data not shown). We then examined the transcriptional and protein levels of ENO2 in different HNSCC cell lines by qRT-PCR and western blotting. Consistently, the levels of ENO2 were stronger in all HNSCC cell lines compared with normal oral keratinocytes (NOK). Moreover, ENO2 was highly expressed in UM-1 and Cal27 cells relative to Cal33 and HSC3 cells (Fig. 1g-h). These findings suggest that ENO2 is likely to be involved in HNSCC development.

### ENO2 drives both cell growth and glucose metabolism in HNSCC cells

**Fig. 2:**
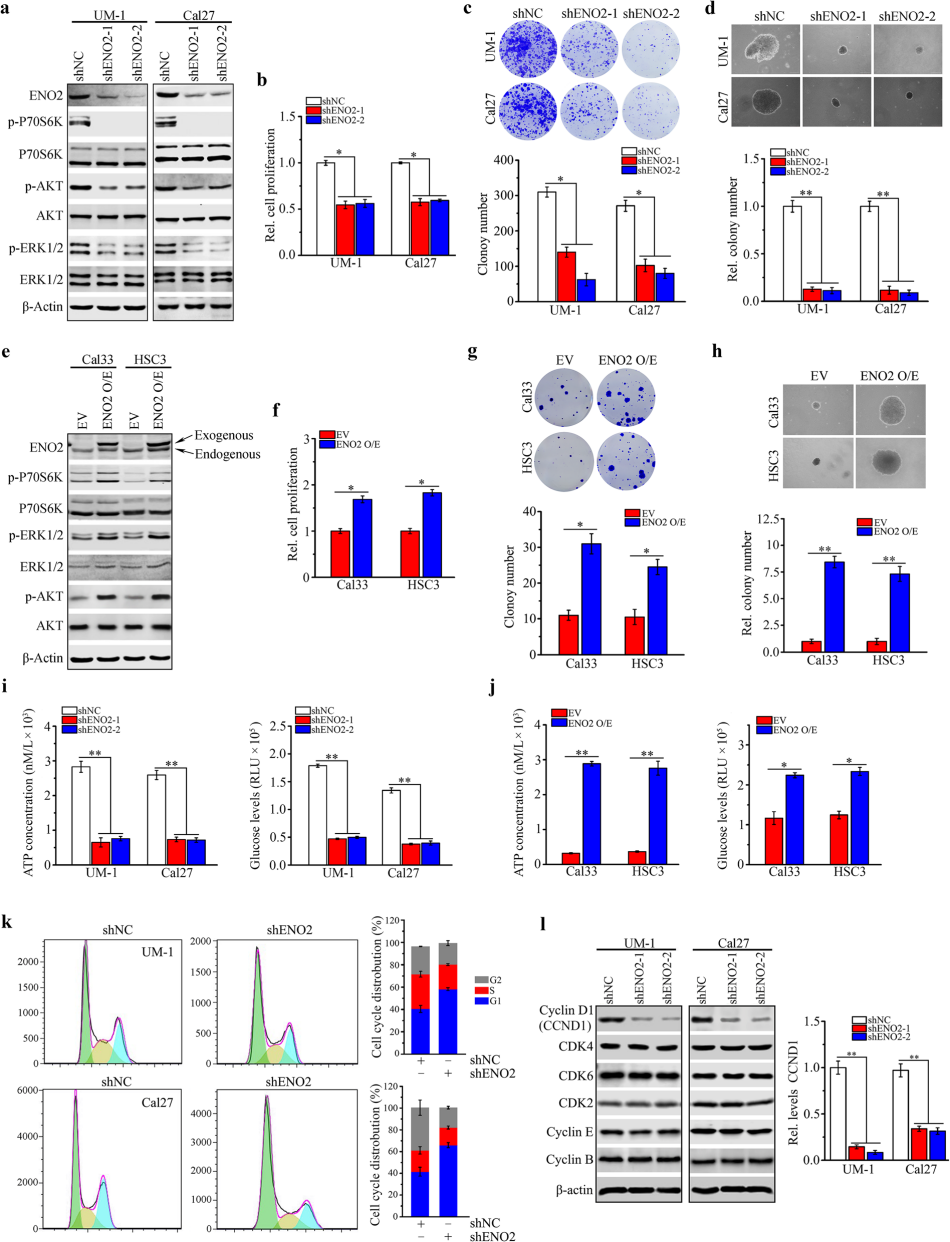
ENO2 promotes cell proliferation and glucose metabolism in HNSCC cells. **a** Effect of ENO2 knockdown on AKT and ERK1/2 signaling pathways in UM-1 and Cal27 cells determined by western blotting. **b**-**d** Effect of ENO2 knockdown on cell proliferation (**b**), colony formation (**c**) and anchorage-independent growth (**d**) in UM-1 and Cal27 cells determined by MTT, clonogenic and soft agar assays, respectively. € Effect of ENO2 overexpression on AKT and ERK1/2 signaling pathways in Cal33 and HSC3 cells determined by western blotting. **f-h** Effect of ENO2 overexpression on cell proliferation (**f**), colony formation (**g**) and anchorage-independent growth (**h**) in Cal33 and HSC3 cells determined by MTT, clonogenic and soft agar assays, respectively.** i **Effect of ENO2 knockdown on the cellular levels of ATP and glucose in UM-1 and Cal27 cells determined by ATP assay kit and Glucose Uptake-Glo assay kit, respectively. **j** Effect of ENO2 overexpression on the cellular levels of ATP and glucose uptake in Cal33 and HSC3 cells determined by ATP assay kit and Glucose Uptake-Glo assay kit, respectively. **k** Effect of ENO2 knockdown on the cell cycle progression in UM-1 and Cal27 cells determined by flow cytometry with PI staining. The quantitative data are shown in the right panel. **l** Effect of ENO2 knockdown on cell cycle-related protein expression in UM-1 and Cal27 cells determined by western blotting. The quantitative data are shown in the right panel. *p<0.05; **p<0.01

We next generated ENO2 knockdown (shENO2-1 and shENO2-2) in ENO2 high-expressing UM-1 and Cal27 cells using lentiviral shRNAs (Fig. 2a). ENO2 knockdown in UM-1 and Cal27 cells significantly inhibited cell proliferation and the capability of cell colony formation and anchorage-independent growth (Fig. 2b-d). Moreover, knockdown of ENO2 remarkably inactivated AKT and ERK1/2 signaling (Fig. 2a), which was consistent with the results reported from other types of cancer [15]. In contrast, overexpression of ENO2 in Cal33 and HSC3 cells with low levels of endogenous ENO2 produced the opposite effects to those seen in ENO2 knockdown UM-1 and Cal27 cells (Fig. 2e-h). Given that ENO2 is a key enzyme in glycolysis, we determined the ATP concentration and glucose levels upon modification of ENO2 gene expression in HNSCC cells. Loss of ENO2 reduced the intracellular ATP levels and glucose consumption of UM-1 and Cal27 cells (Fig. 2i), while overexpression of ENO2 significantly increased the intracellular levels of ATP and glucose in Cal33 and HSC3 cells (Fig. 2j).

Suppression of cell growth in tumors is usually associated with cell cycle arrest. Next, we sought to determine whether ENO2 expression affected cell cycle in HNSCC cells. Relative to knockdown control cells, ENO2 knockdown UM-1 and Cal27 cells were arrested in the G0/G1 phase, accompanied by a decrease in cells in S and G2/M phases (Fig. 2k). We then examined the expression alterations of the key cell cycle-related proteins in ENO2 knockdown and control HNSCC cells. CCND1 is often overexpressed in a variety of cancers and is associated with tumorigenesis and metastasis, and its loss is sufficient to induce G1 cell cycle arrest [25]. Western blotting showed that only CCND1 was downregulated in both UM-1 and Cal27 when ENO2 was genetically depleted (Fig. 2l), suggesting that ablation of ENO2 inhibits cell proliferation at least in part through blocking CCND1-mediated cell cycle progression. Collectively, these findings support the critical role of ENO2 in promoting HNSCC cell growth and glucose metabolism.

### ENO2 knockdown inhibits growth and glucose metabolism of HNSCC cells partially through downregulating PKM2

**Fig. 3:**
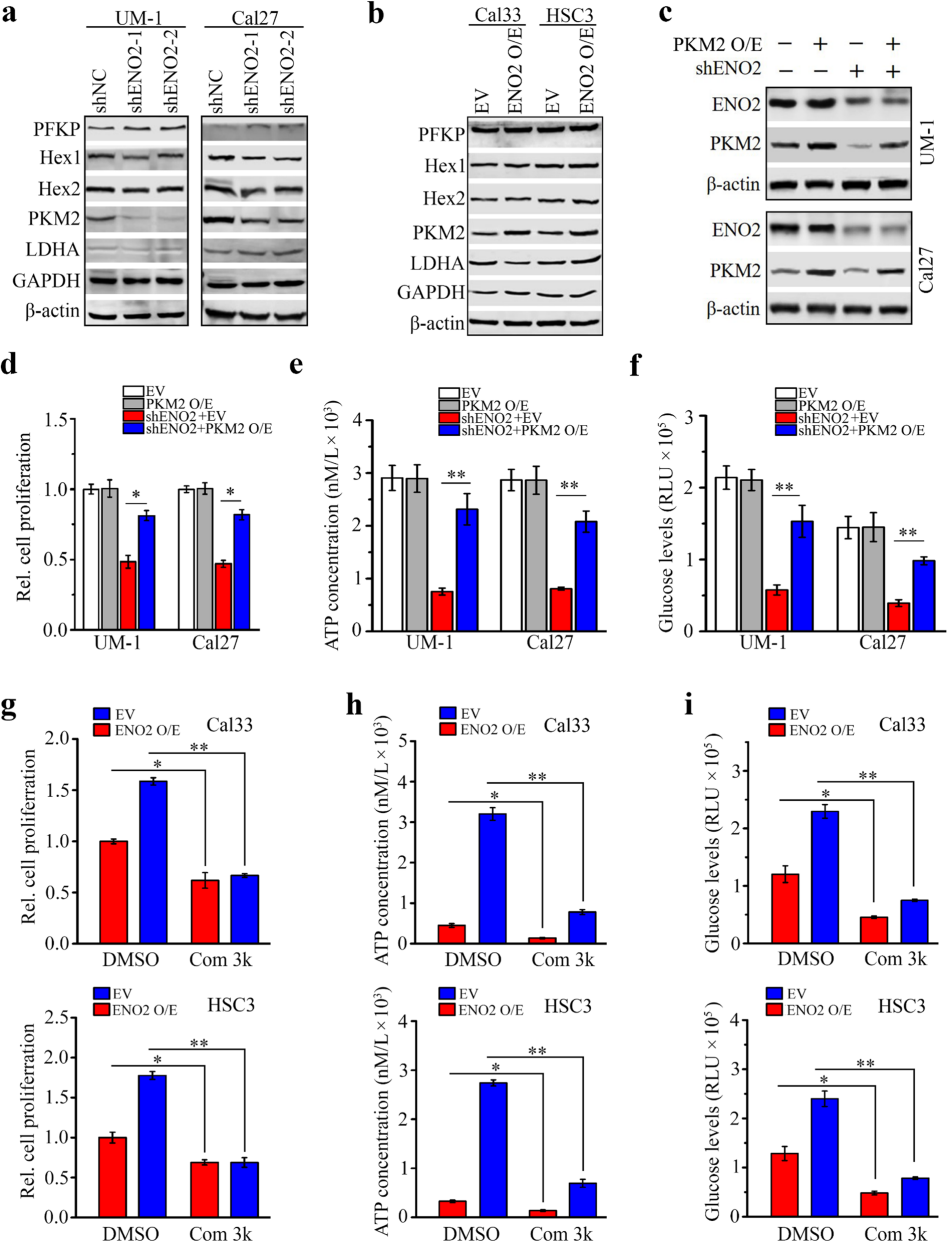
Knockdown of ENO2 inhibits growth and glucose metabolism of HNSCC cells through downregulating PKM2. **a**, **b** Effect of ENO2 knockdown (**a**) and overexpression (**b**) on glycolytic molecules determined by western blotting. **c** Restoration of PKM2 expression in ENO2 knockdown UM-1 and Cal27 cells. **d**-**f** Effect of PKM2 expression restoration on cell proliferation (**d**) and intracellular levels of ATP (**e**) and glucose (**f**) in ENO2 knockdown UM-1 and Cal27 cells. **g** Effect of 5µM compound 3k (Com 3k) on cell proliferation in ENO2 overexpressing (ENO2 O/E) and control Cal33 and HSC3 cells. **h** Effect of 5µM compound 3k on intracellular levels of ATP in ENO2 overexpressing and control Cal33 and HSC3 cells. **i** Effect of 5µM compound 3k on intracellular levels of glucose in ENO2 overexpressing and control Cal33 and HSC3 cells.  *p<0.05; **p<0.01

To explore the downstream targets of ENO2 in HNSCC cells, we determined the changes in metabolism-associated molecules with or without ENO2 loss. These molecular alterations were screened by western blotting with the Glycolysis antibody sampler kit. This analysis showed remarkably reduced PKM2 protein levels in ENO2 knockdown UM-1 and Cal27 cells compared with the knockdown control cells (Fig. 3a). Conversely, increased PKM2 was found in Cal33 and HSC3 cells when ENO2 was overexpressed (Fig. 3b), which confirmed the regulation of PKM2 by ENO2 in HNSCC cells. To explore the function of PKM2 in ENO2-mediated glucose consumption and ATP production, we transfected ENO2 knockdown UM-1 and Cal27 cells with PKM2 overexpressing vector. Ectopic expression of PKM2 restored its levels in ENO2 knockdown cells (Fig. 3c), leading to an increase in cell proliferation and intracellular levels of ATP and glucose uptake compared with ENO2 knockdown cells expressing empty vector (Fig. 3d-f). On the contrary, suppressing PKM2 activity using its selective small molecule inhibitor, compound 3k, attenuated increased cell proliferation, intracellular ATP levels and glucose consumption induced by overexpression of ENO2 in Cal33 and HSC3 cells (Fig. 3g-i). These findings suggest that ENO2 controls growth and glucose metabolism of HNSCC cells at least partially via PKM2.

### AP-III-a4 suppresses ENO2-mediated cell proliferation and glycolysis in HNSCC cells

**Fig. 4:**
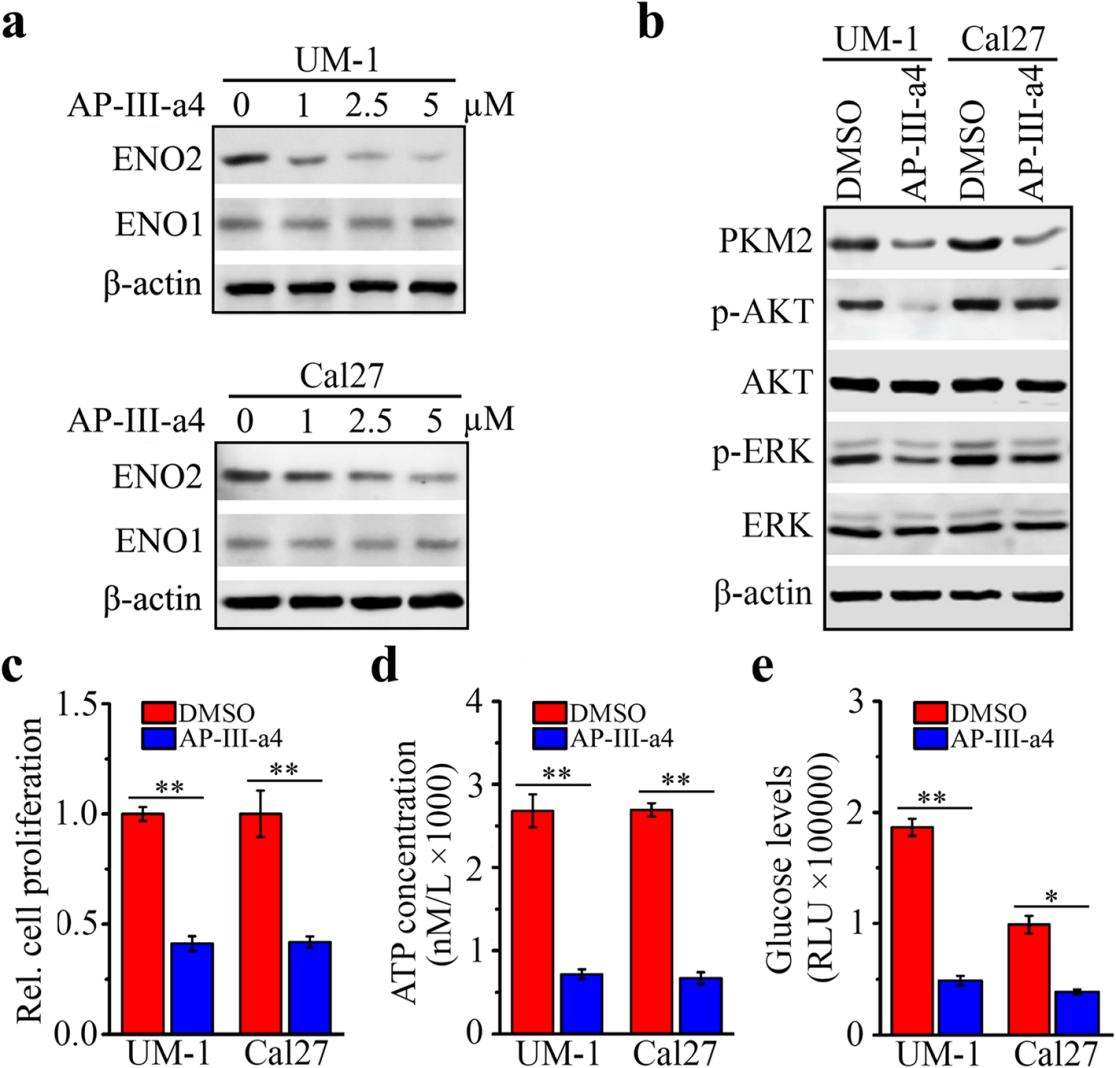
AP-III-a4 inhibits cell proliferation and glucose metabolism in HNSCC cells through suppressing ENO2-mediated downstream signaling. **a** Effect of AP-III-a4 treatment on ENO1 and ENO2 protein levels determined by western blotting. UM-1 and Cal27 cells were treated with AP-III-a4 at a dose range (0, 1, 2.5, 5µM) for 48 h. **b**-**e** Effect of AP-III-a4 treatment on ENO2-mediated downstream targets (**b**), cell proliferation (**c**), and intracellular levels of ATP (**d**) and glucose uptake (**e**). UM-1 and Cal27 cells were treated with or without 5µM AP-III-a4 for 48 h.  *p<0.05; **p<0.01

AP-III-a4, a small molecule enolase inhibitor, is the first non-substrate analogue that directly binds to enolase and inhibits its activity [26]. We treated UM-1 and Cal27 cells with various concentrations of AP-III-a4 for 48 hrs and found that AP-III-a4 inhibited ENO2 expression in a dose-dependent manner (Fig. 4a). However, AP-III-a4 showed no inhibitory effect on ENO1 expression up to 5 µM (Fig. 4a). Western blotting further revealed the on-target effect of AP-III-a4 as evidenced by its inhibitory role in ENO2 downstream signaling, which included decreased PKM2 protein levels and AKT and ERK1/2 phosphorylation levels when UM-1 and Cal27 cells were treated with this inhibitor (Fig. 4b). In addition, significantly reduced cell proliferation, intracellular ATP concentration and glucose uptake levels were seen in UM-1 and Cal27 cells in the presence of AP-III-a4 (Fig. 4c-e).

### ENO2 interacts with PKM2 that prevents PKM2 from ubiquitin-mediated proteasomal degradation

**Fig. 5:**
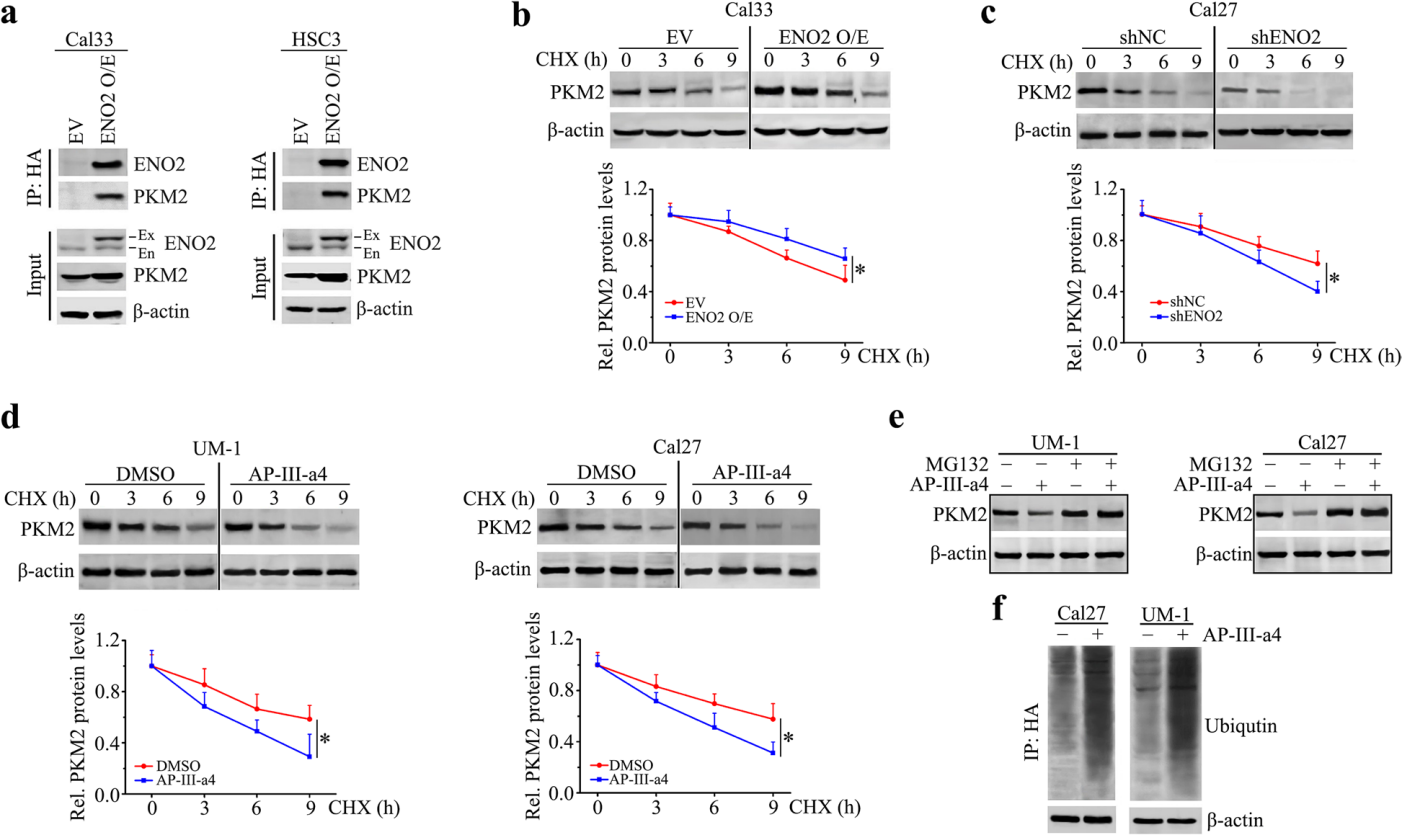
ENO2 interacts with PKM2 and protects it from ubiquitin-mediated proteasomal degradation in HNSCC cells. **a** The binding of ENO2 to PKM2 in ENO2 overexpressing Cal33 and HSC3 determined by IP with anti-HA antibody. **b**, **c** Effect of ENO2 overexpression (**b**) or knockdown (**c**) on the half-life of PKM2 protein determined by CHX chase assays. ENO2 overexpressing or knockdown cells were treated with 100 μg/ml CHX for the indicated hours. **d** Effect of AP-III-a4 on the half-life of PKM2 protein determined by CHX chase assays. Cal27 and UM-1 cells were treated with 100μg/ml CHX for the indicated hours in the presence or absence of 5µM AP-III-a4. **e** Effect of AP-III-a4 on PKM2 protein levels in the presence or absence of MG132. Cal27 and UM-1 cells were pretreated with 10μM MG132 for 4 h and then treated with 5µM AP-III-a4 for 24 h. **f** Effect of AP-III-a4 on PKM2 ubiquitination. PKM2 overexpressing Cal27 and UM-1 cells were pretreated with 10μM MG132 for 4 h before treatment of 5µM AP-III-a4. Cell lysates were IP using anti-HA antibody and immunoblotted with anti-ubiquitin antibody. β-Actin immunoblotting on total lysate is shown to normalize the input

Surprisingly, qRT-PCR showed that ENO2 knockdown did not decrease mRNA levels of PKM2 in UM-1 and Cal27 cells (data not shown), suggesting ENO2 may regulate PKM2 expression at protein level. To address this, we first determined the potential interaction between ENO2 and PKM2 proteins by immunoprecipitating ENO2 protein with anti-HA antibody in ENO2 overexpressing Cal33 and HSC3 cells. IP assay identified PKM2 in the ENO2 immunocomplex (Fig. 5a). We then treated ENO2 overexpressing and control Cal33 cells with CHX to block *de novo* protein synthesis and collected cell lysates at different time points. Western blotting showed that overexpression of ENO2 prolonged the half-life of PKM2 proteins compared with control cells (Fig. 5b). In contrast, knockdown of ENO2 in Cal27 cells resulted in a dramatic decrease in the half-life of PKM2 (Fig. 5c). Using CHX chase assay, we found that PKM2 was degraded much faster in AP-III-a4-treated UM-1 and Cal27cells than in control cells exposed to DMSO (Fig. 5d). Moreover, the presence of the proteasome inhibitor, MG132, largely rescued the reduction of PKM2 in the presence of AP-III-a4 (Fig. 5e). These observations indicate that loss of ENO2 destabilizes PKM2 by prompting its proteasomal degradation. To determine whether modulation of ENO2 expression could alter the ubiquitination of PKM2 and consequently its protein stability, Cal27 cells transfected with expression vectors containing HA-tagged PKM2 were pretreated MG132 and subsequently treated with AP-III-a4. Immunoprecipitation of anti-HA antibody followed by ubiquitin immunoblotting analysis revealed that inhibition of ENO2 increased the fraction of ubiquitinated PKM2 (Fig. 5f). These results suggest that ENO2 binds to PKM2 that prevents PKM2 from ubiquitin-mediated proteasomal degradation.

### ENO2 regulates PKM2 to drive CCND1-mediated cell cycle progression in HNSCC cells

**Fig. 6:**
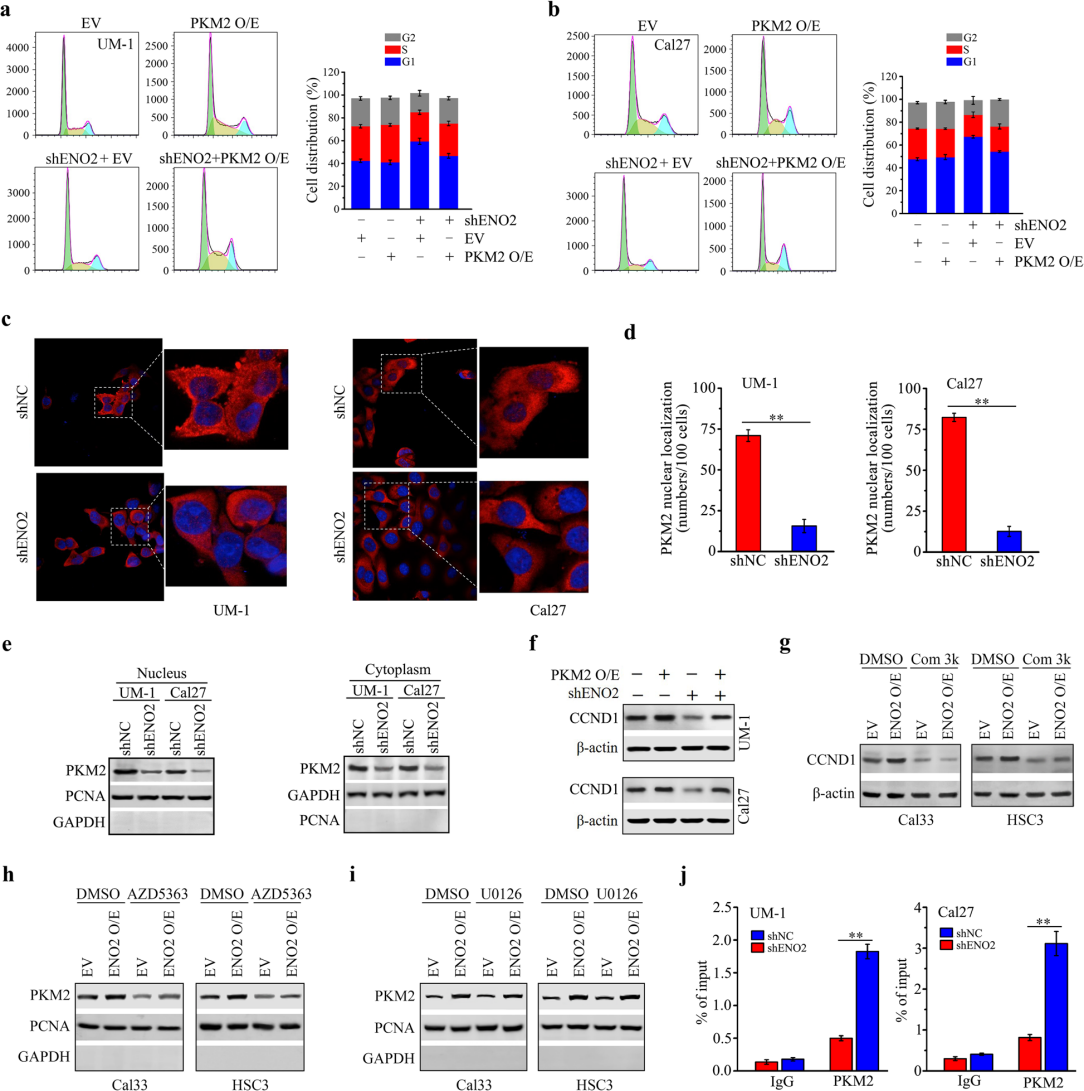
ENO2 regulates CCND1-mediated cell cycle progression *via* PKM2 in HNSCC cells. **a**, **b** Effect of PKM2 expression restoration on cell cycle in ENO2 knockdown UM-1 and Cal27 cells. **c**, **d** The nuclear translocation of PKM2 in ENO2 knockdown and control UM-1 and Cal27 cells. Representative confocal images and quantitative data from 100 cells are shown in (**c**) and (**d**), respectively. **e** Effect of ENO2 knockdown on nuclear and cytoplasmic PKM2 protein levels in UM-1 and Cal27 cells. **f** Effect of PKM2 expression restoration on CCND1 expression in ENO2 knockdown UM-1 and Cal27 cells. **g** Effect of compound 3k (Com 3k) on CCND1 expression in ENO2 overexpressing (ENO2 O/E) and control Cal33 and HSC3 cells. **h** Effect of AZD5363 on nuclear PKM2 protein levels in ENO2 overexpressing and control Cal33 and HSC3 cells. **i** Effect of U0126 on nuclear PKM2 protein levels in ENO2 overexpressing and control Cal33 and HSC3 cells. In (**h**-**i**), the purity of the nuclear fractions was indicated by the nuclear PCNA protein and the absence of cytosolic GAPDH protein. **j** The binding of PKM2 protein on the CCND1 gene promoter in ENO2 knockdown and control UM-1 and Cal27 cells determined by ChIP-qPCR assays. **p<0.01

Besides the well-known role of PKM2 in glycolysis, it has nonmetabolic functions in controlling cell cycle progression and tumorigenesis [27, 28]. Based on the above experimental findings, we further examined the contribution of PKM2 to ENO2-mediated cell cycle progression. Restoration of PKM2 expression in ENO2 knockdown UM-1 and Cal27 cells remarkably reversed ENO2 loss-mediated G1 cell cycle arrest (Fig. 6a, b), supporting the notion that PKM2 is the key molecule in ENO2-mediated cell division. Increasing evidence demonstrates that nuclear PKM2 directly regulates cell cycle progression [29–31]. We then preformed IF assay to determine the cellular localization of PKM2 in the cytoplasm and nucleus, which showed that ENO2 knockdown dramatically blocked PKM2 aggregation in the nucleus (Fig. 6c, d). To corroborate this, we isolated nuclear and cytosolic fragments from ENO2 knockdown and control HNSCC cells, followed by western blotting. This analysis showed that both cytosolic and nuclear PKM2 were reduced in UM-1 and Cal27 cells upon ENO2 knockdown, but more remarkable decrease in PKM2 was observed in the nucleus (Fig. 6e).

As CCND1 is a downstream target of nuclear PKM2, we sought to determine CCND1 expression in ENO2 knockdown HNSCC cells with or without PKM2 expression restoration. Western blotting showed that CCND1 levels were rescued in ENO2 knockdown UM-1 and Cal27 cells when PKM2 expression was restored (Fig. 6f). Conversely, CCND1 expression was suppressed in the presence of PKM2 inhibitor compound 3k regardless of ENO2 overexpression in Cal33 and HSC3 cells (Fig. 6g), indicating the involvement of PKM2 in ENO2-mediated CCND1 regulation.

AKT and ERK1/2 signaling pathways have been reported to be involved in PKM2 nuclear translocation [32, 33], and were activated by ENO2 in HNSCC cells (Fig. 2a and e). We next determined which pathway contributed to the nuclear translocation of PKM2 in the context of ENO2 signaling. For this purpose, ENO2 overexpressing and control Cal33 and HSC3 cells were treated with AKT inhibitor AZD5363 and MEK inhibitor U0126, respectively. Western blotting analysis of nuclear protein extracts showed that AZD5363, but not U0126, dramatically suppressed the nuclear levels of PKM2 regardless of ENO2 overexpression (Fig. 6h, i and Supplementary Fig. S1), suggesting the contribution of AKT signaling to ENO2-mediated PKM2 nuclear translocation. To investigate whether the specific binding of PKM2 to the CCND1 gene promoter was required for the transcriptional activation of CCND1, we tested the transcriptional regulatory relationship between PKM2 and CCND1 by ChIP-qPCR assay. We found that the amount of PKM2 bound to the CCND1 promoter was significantly reduced in UM-1 and Cal27 cells when ENO2 was knocked down (Fig. 6j), demonstrating a strong correlation of PKM2 occupancy with CCND1 expression.

### AP-III-a4 exhibits a strong inhibitory effect on head and neck tumors in xenograft mice

**Fig. 7:**
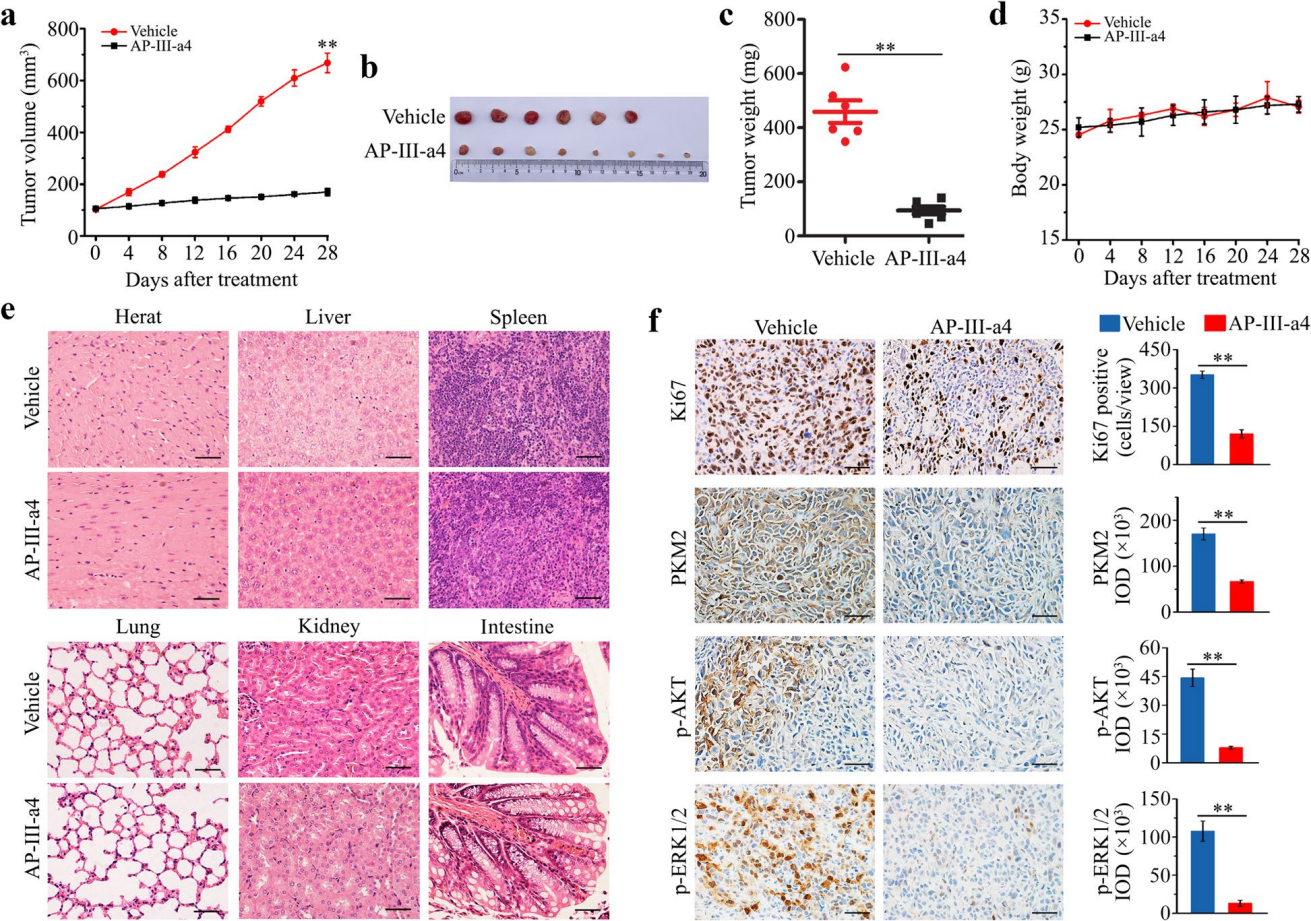
AP-III-a4 induces remission of head and neck tumors in xenograft mice. **a** Effect of AP-III-a4 treatment on tumor growth measured by tumor volume at the indicated time points. Six-week-old nude mice with subcutaneously implanted UM-1 cells were randomized into two groups to receive vehicle (PBS) or AP-III-a4 treatment for 28 days. **b** Images of xenograft tumors of each group at the same time of study end. **c** Tumor weight measured at the end of the treatment. **d** Mouse body weight recorded every four days during the treatment process. **e** Histology of major organs from mice receiving vehicle or AP-III-a4 treatment. **f** Immunostaining of Ki67, PKM2, p-ERK and p-AKT in tumor xenografts from mice receiving vehicle or AP-III-a4 treatment. Representative IHC images and quantitative data are shown in the left and right panels, respectively. Scale bar depicts 2mm. *p<0.05; **p<0.01

To investigate the therapeutic efficacy of AP-III-a4 in HNSCC, nude mice bearing UM-1-derived xenografts were established. When tumors reached a volume of 100 mm^3^, 20 mg/kg AP-III-a4 was given by intraperitoneal injection every other day for 28 days. In line with the results from cell culture, tumor burden was reduced in AP-III-a4 treated mice as illustrated by smaller tumor size and decreased tumor weight compared with the group receiving vehicle (Fig. 7a-c). To determine the safety of AP-III-a4 administration, we measured the body weight and major organ histopathology of each treatment group. There was no significant difference in mouse body weight or morphological changes in the major organs between mice receiving vehicle or AP-III-a4 treatment (Fig. 7d. e), indicating that AP-III-a4 used in this study did not produce detectable systemic toxicities. (Fig. 7f). Western blotting data showed that the expression of ENO2, but not ENO1, was repressed by AP-III-a4 in tumor tissues (Supplementary Fig. S2a), which was confirmed by IHC (Supplementary Fig. S2b). To further evaluate the on-target effect of in vivo treatments, xenograft tissue sections were immunostained with antibodies against ENO2 downstream targets, including PKM2, p-AKT and p-ERK1/2. Consistent with the in vitro data, the protein levels of PKM2 and the phosphorylation levels of AKT and ERK1/2 were significantly reduced in xenografts from mice treated with AP-III-a4 when compared with vehicle ​(Fig. 7f). Moreover, IHC analysis showed decreased Ki67-positive tumor cells when treated with AP-III-a4 ​(Fig. 7f), which confirmed the inhibitory effect of this drug on tumor growth. Taken together, our study provides evidence that in the xenograft mouse model, targeting ENO2 with AP-III-a4 represents a safe and effective way to constrain HNSCC.

## Discussion

**Fig. 8:**
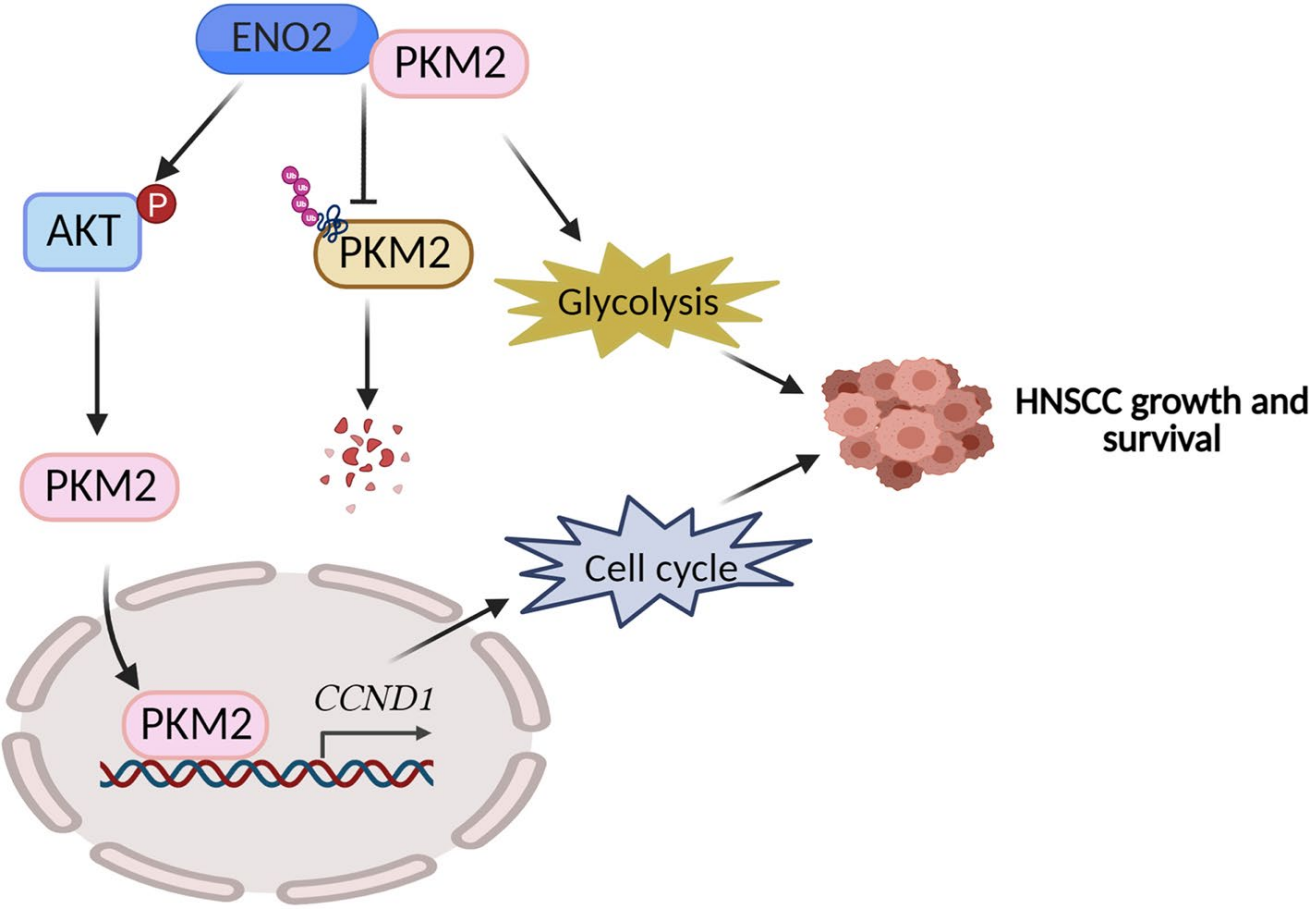
Proposed model for PKM2 regulation by ENO2 in HNSCC cells. ENO2 binds to PKM2 to prevent PKM2 protein degradation by the ubiquitin-proteasome system and facilitates the nuclear translocation of PKM2 by mediating AKT phosphorylation, potentiating PKM2-mediated glycolytic flux and CCND1-associated cell cycle progression

HNSCC is highly aggressive and multi-factorial disease in the upper aerodigestive tract and uncontrolled proliferation is the main cause of death in HNSCC patients [1, 2]. Therefore, elucidating the mechanisms underpinning uncontrolled cancer cell proliferation will provide new theoretical platforms and potential therapeutic targets for the treatment of HNSCC. Accumulating evidence has shown that cancer cells engage in a metabolic program to enhance biosynthesis and support cell proliferation, survival and long-term maintenance [11, 34]. Like other solid tumors, HNSCC shows high glycolysis to meet its metabolic requirements [35], suggesting that glucose metabolism is closely related to the occurrence and development of HNSCC. ENO2 is a glycolytic enzyme to be required for tumorigenesis and malignancy [12, 15]; however, its role HNSCC development has not been determined previously. In the present study, we found that ENO2 genetic depletion markedly suppressed HNSCC cell proliferation and targeting it with the ENO inhibitor AP-III-a4 displayed a strong anti-HNSCC effect in preclinical animal models. PKM2 is another key glycolytic enzyme catalyzing the last step of glycolysis to yield adenosine ATP and pyruvate, which is commonly more abundant in highly proliferating cancer cells [36]. In HNSCC cells, we demonstrate that ENO2 not only facilitates glucose consumption by stabilizing PKM2 proteins but also promotes its nuclear translocation to enhance CCND1-dependent cell cycle progression (Fig. 8). These two mechanisms synergically potentiate HNSCC cell growth and survival (Fig. 8). To our knowledge, this is the first study illustrating the mechanistic link between ENO2 and PKM2 in cancer cells.

ENO1 (ENO-α), ENO2 (ENO-γ) and ENO3 (ENO-β) are three enolase isoforms in mammalian cells, catalyzing the transformation of 2-PG to PEP during glycolysis. ENO1, also referred to as 2-phospho-D-glycerate hydrolase, is a multifunctional oncoprotein that is present both at the cell surface and in the cytoplasm, contributing to it displaying seven out of ten “hallmarks of cancer” [37, 38]. Many studies have demonstrated that the localization and overexpression of ENO1 on the cancer cell surface make it a potential prognostic and diagnostic cancer biomarker as well as an accessible oncotarget [39, 40]. Using TCGA HNSCC database, we also found upregulated ENO1 in HNSCC tissues relative to normal counterparts (Supplementary Fig. S3a). However, immunostaining with anti-ENO1 antibody did not show significant increase of ENO1 levels in the primary HNSCC tissues examined compared with normal tissue samples (data not shown), supporting ENO2 as a more favorable target than ENO1. ENO3 is predominantly expressed in adult striated muscle, including skeletal and cardiac muscle [41]. Specially, ENO3 is considered an STK11 mutation-specific target as overexpression of this isoform is the direct consequence of STK11 loss-of-function mutation [42]. Unlike ENO1 and ENO2, the expression of ENO3 is significantly downregulated in HNSCC cells (Supplementary Fig. S3b). Further studies show that ENO1 and ENO3 were not correlated with HNSCC prognosis based on TCGA analysis results (Supplementary Fig. S4). This is the main reason for our research focus on the role of ENO2 in HNSCC development.

Cell metabolism is the set of chemical reactions organized into metabolic pathways. Enzymes are crucial to metabolism, allowing the fine regulation of metabolic pathways to maintain a constant set of conditions in response to changes in the cell’s environment. The glycolysis pathway and its enzyme genes are highly conserved, playing critical roles in physiological and pathological processes [11, 21]. ENO2 is a cytoplasmic glycolytic enzyme that plays its own role in glycolysis. However, our study does not differentiate the alterations in glucose uptake and ATP production in ENO2 knockdown HNSCC cells are resulted from ENO2 loss itself or downregulated PKM2. The possible strategy to tackle this issue is to dissect ENO2- and PKM2-involved steps during the glycolytic process. It is worth noting that ENO2 binds to PKM2 and prevents it from ubiquitin-mediated proteasomal degradation, but whether PKM2 degradation upon ENO2 loss is through mono- or poly-ubiquitination is still unclear. Therefore, understanding the mechanism of ubiquitination in PKM2 degradation will allow us to develop new approaches to perturb this pathway for constraining ENO2-driven HNSCC.

PKM2 functions as a metabolic enzyme, a protein kinase, or a transcriptional coactivator of genes that influence cancer cell proliferation [19, 20, 43]. A recent study has revealed elevated expression and phosphor-activation (Tyr105) of PKM2 in HNSCC cell lines from different origins, including laryngopharynx, pharynx-derived metastasis from pleural effusion, soft palate-derived metastasis from a cervical lymph node, alveolar ridge, and HPV(+) and HPV(-) tongues [44]. Our bioinformatics analysis of TCGA database clearly shows upregulated PKM2 expression in HNSCC tissues compared with normal tissues (Supplementary Fig. S5), raising the possibility of PKM2 targeting as a promising strategy for HNSCC treatment. To date, several PKM2-inhibitory compounds have been developed. For example, shikonin is an active chemical component extracted from *Lithospermum erythrorhizon*, which shows promise in reducing PKM2-mediated cancer cell glycolysis [45]. Compound 3k used in our study has been reported to display more potent PKM2 inhibitory activity than the reported optimal PKM2 inhibitor shikonin [46]. Consistent with the previous study [44], compound 3k treatment resulted in cytotoxicity in all HNSCC cell lines examined through inhibiting PKM2 function. Interestingly, several small molecules that selectively activate PKM2 over other pyruvate kinase isoforms, such as DASA-58 and TEPP-46, were also discovered as an anticancer therapeutic strategy by inducing tetramerization of the kinase and suppressing lactate secretion[47, 48]. Here, we show that depletion of ENO2 induces HNSCC remission through impairing metabolic and nonmetabolic roles of PKM2, providing new evidence that targeting PKM2 in HNSCC cells could be a therapeutic option to further investigate.

Interestingly, PFKP, another glycolytic enzyme that catalyzes the rate-limiting step of glycolysis by converting fructose 6-phosphate and ATP to fructose-1,6-bisphosphate and ADP [49, 50], was upregulated in ENO2 knockdown UM-1 and Cal27 cells. Increased PFKP could be one of the bypass signaling activations to maintain the low levels of glucose metabolism in ENO2-deficient cancer cells. However, there is no report on the relationship between ENO2 and PFKP, which remains to be determined. In addition, ENO2 loss produces cell context-dependent phenotypes, such as reduced LDHA in UM-1 while increased LDHA in Cal27 upon ENO2 knockdown. LDHA has a key role in tumor metabolism, but how malignant cells adapt to LDHA inhibition remains unclear [51]. It seems that the regulation of LDHA by ENO2 may be dependent on the genetic background of HNSCC cells, which warrants further investigation. Furthermore, our data shows a significant correlation between ENO2 expression and clinical N grade. N describes the involvement of lymph nodes near the primary tumor, suggesting ENO2 may promote glycolysis to produce more ATP and facilitate cell division and eventually support HNSCC cells for lymph node metastasis. Another implication is that ENO2 may play a critical role in mediate the immune response. Although we are still far from the clinical use of ENO2 as a prognostic biomarker for HNSCC patients, it is worth focusing efforts on fully understanding the function of ENO2 at a whole-body level.

Inhibiting enolase appears a promising approach to eradicating tumors, as intensive studies have demonstrated that enolase affects the glycolysis pathway during tumor development and acts as a “moonlighting” protein with important roles in diverse cellular processes that are not related to its function in glycolysis [52]. AP-III-a4 is the first reported enolase inhibitor that is suitable for biological assays [53]. AP-III-a4 was developed as a probe to study the non-glycolytic functions of enolase, which has potential for therapeutic development to treat type 2 diabetes mellitus (T2DM) [54, 55]. We show for the first time that AP-III-a4 has great potential to suppress HNSCC growth and development. In the present study, we surprisingly found a dose-dependent suppressive effect of this compound on ENO2 expression, without affecting ENO1 expression, which may explain the high selectivity of AP-III-a4 for ENO2. The compound showing specificity to ENO2 but not ENO1 turned to be discrepant to the other studies that used it as an ENO1 inhibitor [26]. However, there is a big possibility that increasing the treatment dose of AP-III-a4 may lead to a reduction in both ENO1 and ENO2. It is still unclear about the efficacy of AP-III-a4 in enolase-mediated glycolysis function in our preclinical animal models, and more specific investigations should be approached to show evidence of the *in vivo* impact regarding the ENO2 functions. Recently, another potent ENO2-specific inhibitor of enolase known as POMHEX has gained attention. This inhibitor is a racemic mixture and a cell-permeable pivaloyloxymethyl (POM) prodrug of HEX, which can selectively kill ENO1-deleted glioma cells at low-nanomolar concentrations and eradicate intracranial orthotopic ENO1-deleted tumors in mice at doses well-tolerated in non-human primates [56]. It would be interesting to determine the therapeutic efficacy of POMHEX in HNSCC tumors harboring high levels of ENO2, which also merits further comparison with AP-III-a4. As long-established cell lines could not represent clinical diseases, one of our follow-up studies will be to improve clinical significance by evaluating the therapeutic benefits of AP-III-a4 in HNSCC patient-derived xenograft models.

## Conclusions

In summary, our findings suggest that ENO2 is a novel druggable target in HNSCC as blocking its function could strongly impair cancer cell proliferation due to reduced glycolysis and cell cycle arrest. The knowledge gained from this study may provide a scientific basis for developing ENO2-based targeted therapy to improve the care of patients with HNSCC.

## Supplementary Information

**Additional file 1:** **Supplementary Figure S1. **Quantitative data of relative nuclear PKM2 protein levels in ENO2 overexpressing and control Cal33 and HSC3 cells in the presence of AZD5363 (a) or U0126 (b). Representative images of western blot are shown in Figure 6h and 6i. *p<0.05.

**Additional file 2:** **Supplementary Figure S2. **AP-III-a4 represses ENO2 protein levels in head and neck tumors in xenograft mice. (a) Effect of AP-III-a4 on ENO1 and ENO2 protein levels in mouse xenograft tumors measured by western blotting. (b) Immunostaining of ENO1 and ENO2 in tumor xenografts from mice receiving vehicle or AP-III-a4 treatment. Representative IHC images and quantitative data are shown in the left and right panels, respectively. Scale bar depicts 2mm. *p<0.05; **p<0.01.

**Additional file 3:** **Supplementary Figure S3. **The expression of ENO1 (a) and ENO3 (b) in tumor and normal tissue samples based on TCGA cohort with 33 cancer types.

**Additional file 4:** **Supplementary Figure S4. **Kaplan-Meier survival analysis for the correlation between the expression levels of ENO1 (a) and ENO3 (b) and the survival in HNSCC patients.

**Additional file 5:** **Supplementary Figure S5. **The expression of PKM2 in tumor and normal tissue samples based on TCGA database. (**a**) Analysis of PKM2 expression in normal and tumor tissues using TCGA cohort with 33 cancer types. (**b**) Analysis of PKM2 expression in head and neck normal and tumor tissues using TCGA HNSC cohort.

## Data Availability

All data generated or analyzed during this study are included in this published article [and its supplementary information files].

## Abbreviations

CCND1: Cyclin D1
CHX: Cycloheximide
ENO2: Eenolase 2
FDG: Fluoro-2-deoxy-D-glucose
GAPDH: Glyceraldehyde-3-phosphate dehydrogenase
HNSCC: Head and neck squamous cell carcinoma
LDHA: Lactate dehydrogenase A
HK I: Hexokinase I
HK II: Hexokinase II
HPV: Human papillomavirus
NSE: Neuron-specific enolase
2-PG: 2-phosphoglycerate
PEP: Phosphoenolpyruvate
PDAC: Pancreatic ductal adenocarcinoma
PDH: Pyruvate dehydrogenase
PFKP: Phosphofructokinase, platelet
POM: Permeable pivaloyloxymethyl
SCLC: Small-cell lung cancer
T2DM: Type 2 diabetes mellitus
