# Supplementary material for: Mediation of PKM2-dependent glycolytic and non-glycolytic pathways by ENO2 in head and neck cancer development
Source: J Exp Clin Cancer Res. 2023 Jan 2;42:1. doi: 10.1186/s13046-022-02574-0 (PMC9806895; doi:10.1186/s13046-022-02574-0)

**Supporting Information for**

**Mediation of PKM2-dependent glycolytic and non-glycolytic pathways by ENO2 in head and neck cancer development**

**This PDF file includes:**

**Supplementary Figures and Figure legends**

**Supplementary Figure S1.** Quantitative data of relative nuclear PKM2 protein levels in ENO2 overexpressing and control Cal33 and HSC3 cells in the presence of AZD5363 (a) or U0126 (b). Representative images of western blot are shown in Figure 6h and 6i. **p*<0.05.


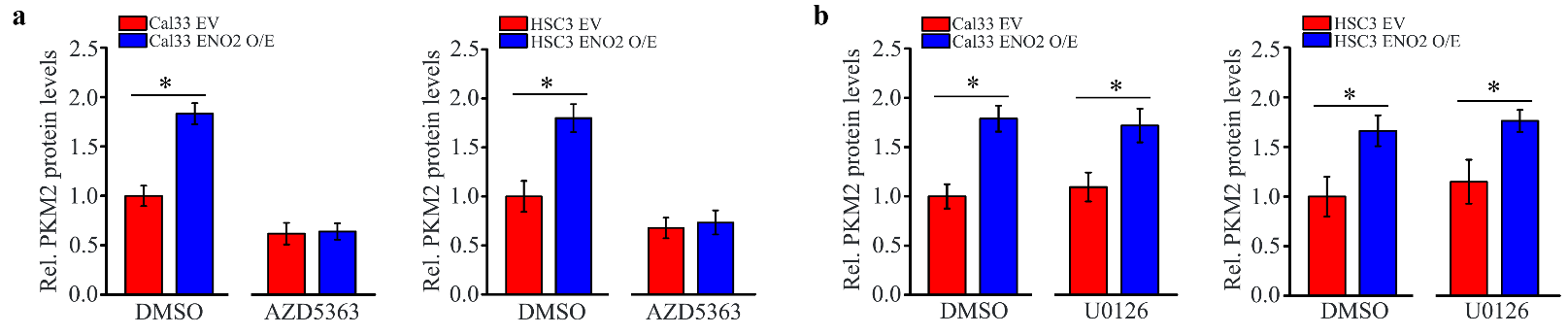

Supplement: Supplementary file 1 — Additional file 1: Supplementary Figure S1. Quantitative data of relative nuclear PKM2 protein levels in ENO2 overexpressing and control Cal33 and HSC3 cells in the presence of AZD5363 (a) or U0126 (b). Representative images of western blot are shown in Figure 6h and 6i. *p<0.05. [file 13046_2022_2574_MOESM1_ESM.docx]
