# Supplementary material for: Mediation of PKM2-dependent glycolytic and non-glycolytic pathways by ENO2 in head and neck cancer development
Source: J Exp Clin Cancer Res. 2023 Jan 2;42:1. doi: 10.1186/s13046-022-02574-0 (PMC9806895; doi:10.1186/s13046-022-02574-0)

**Supporting Information for**

**Mediation of PKM2-dependent glycolytic and non-glycolytic pathways by ENO2 in head and neck cancer development**

**This PDF file includes:**

**Supplementary Figures and Figure legends**

**Supplementary Figure S2.** AP-III-a4 represses ENO2 protein levels in head and neck tumors in xenograft mice.

(a) Effect of AP-III-a4 on ENO1 and ENO2 protein levels in mouse xenograft tumors measured by western blotting. (b) Immunostaining of ENO1 and ENO2 in tumor xenografts from mice receiving vehicle or AP-III-a4 treatment. Representative IHC images and quantitative data are shown in the left and right panels, respectively. Scale bar depicts 2mm. **p*<0.05; ***p*<0.01.


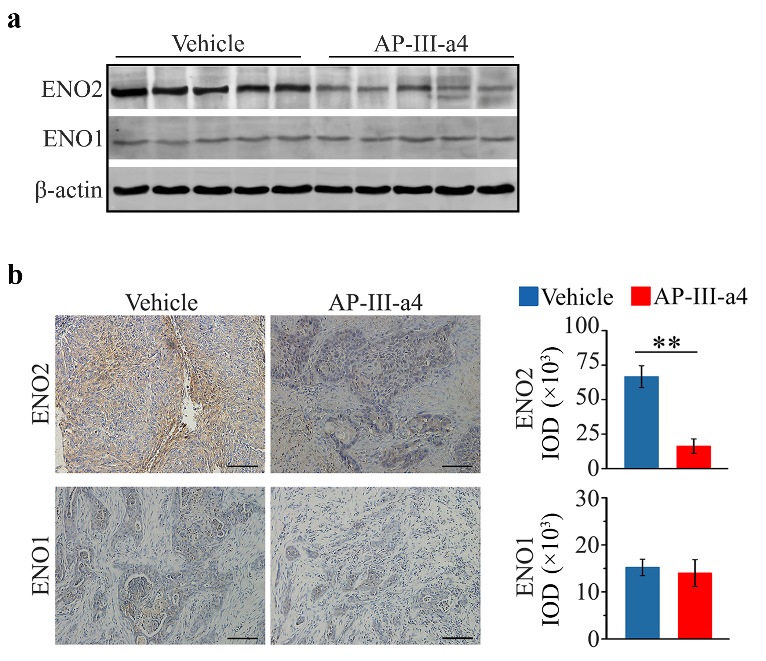

Supplement: Supplementary file 2 — Additional file 2: Supplementary Figure S2. AP-III-a4 represses ENO2 protein levels in head and neck tumors in xenograft mice. (a) Effect of AP-III-a4 on ENO1 and ENO2 protein levels in mouse xenograft tumors measured by western blotting. (b) Immunostaining of ENO1 and ENO2 in tumor xenografts from mice receiving vehicle or AP-III-a4 treatment. Representative IHC images and quantitative data are shown in the left and right panels, respectively. Scale bar depicts 2mm. *p<0.05; **p<0.01. [file 13046_2022_2574_MOESM2_ESM.docx]
