# Supplementary material for: Mediation of PKM2-dependent glycolytic and non-glycolytic pathways by ENO2 in head and neck cancer development
Source: J Exp Clin Cancer Res. 2023 Jan 2;42:1. doi: 10.1186/s13046-022-02574-0 (PMC9806895; doi:10.1186/s13046-022-02574-0)

**Supporting Information for**

**Mediation of PKM2-dependent glycolytic and non-glycolytic pathways by ENO2 in head and neck cancer development**

**This PDF file includes:**

**Supplementary Figures and Figure legends**

**Supplementary Figure S3.** The expression of ENO1 (a) and ENO3 (b) in tumor and normal tissue samples based on TCGA cohort with 33 cancer types.


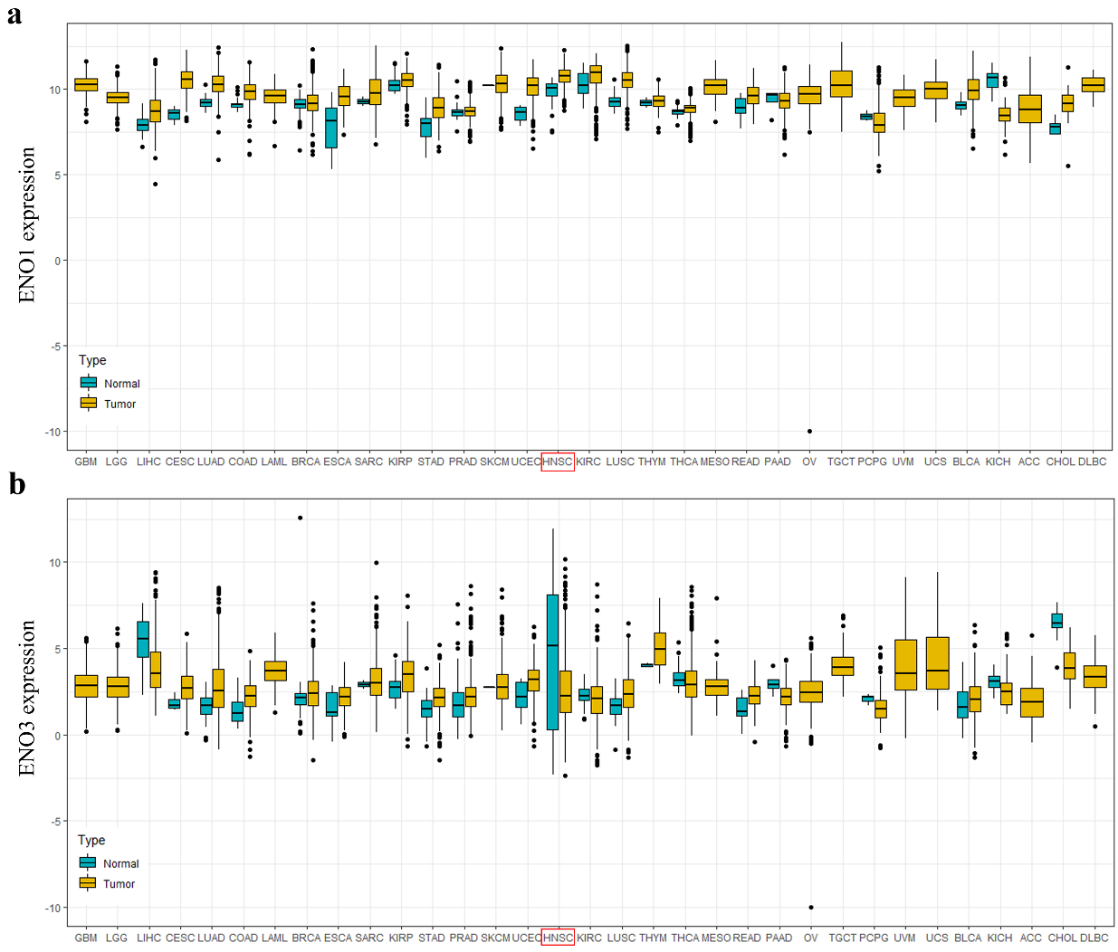

Supplement: Supplementary file 3 — Additional file 3: Supplementary Figure S3. The expression of ENO1 (a) and ENO3 (b) in tumor and normal tissue samples based on TCGA cohort with 33 cancer types. [file 13046_2022_2574_MOESM3_ESM.docx]
