# Supplementary material for: Mediation of PKM2-dependent glycolytic and non-glycolytic pathways by ENO2 in head and neck cancer development
Source: J Exp Clin Cancer Res. 2023 Jan 2;42:1. doi: 10.1186/s13046-022-02574-0 (PMC9806895; doi:10.1186/s13046-022-02574-0)

**Supporting Information for**

**Mediation of PKM2-dependent glycolytic and non-glycolytic pathways by ENO2 in head and neck cancer development**

**This PDF file includes:**

**Supplementary Figures and Figure legends**

**Supplementary Figure S4.** Kaplan-Meier survival analysis for the correlation between the expression levels of ENO1 (a) and ENO3 (b) and the survival in HNSCC patients.


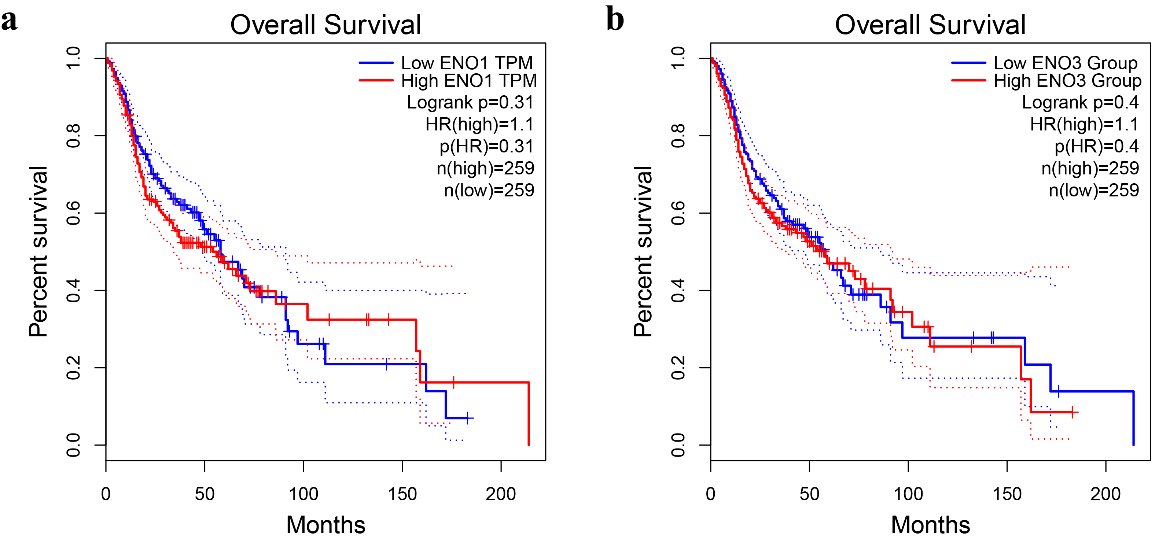

Supplement: Supplementary file 4 — Additional file 4: Supplementary Figure S4. Kaplan-Meier survival analysis for the correlation between the expression levels of ENO1 (a) and ENO3 (b) and the survival in HNSCC patients. [file 13046_2022_2574_MOESM4_ESM.docx]
