# Supplementary material for: Mediation of PKM2-dependent glycolytic and non-glycolytic pathways by ENO2 in head and neck cancer development
Source: J Exp Clin Cancer Res. 2023 Jan 2;42:1. doi: 10.1186/s13046-022-02574-0 (PMC9806895; doi:10.1186/s13046-022-02574-0)

**Supporting Information for**

**Mediation of PKM2-dependent glycolytic and non-glycolytic pathways by ENO2 in head and neck cancer development**

**This PDF file includes:**

**Supplementary Figures and Figure legends**

**Supplementary Figure S5.** The expression of PKM2 in tumor and normal tissue samples based on TCGA database. (a) Analysis of PKM2 expression in normal and tumor tissues using TCGA cohort with 33 cancer types. (b) Analysis of PKM2 expression in head and neck normal and tumor tissues using TCGA HNSC cohort.


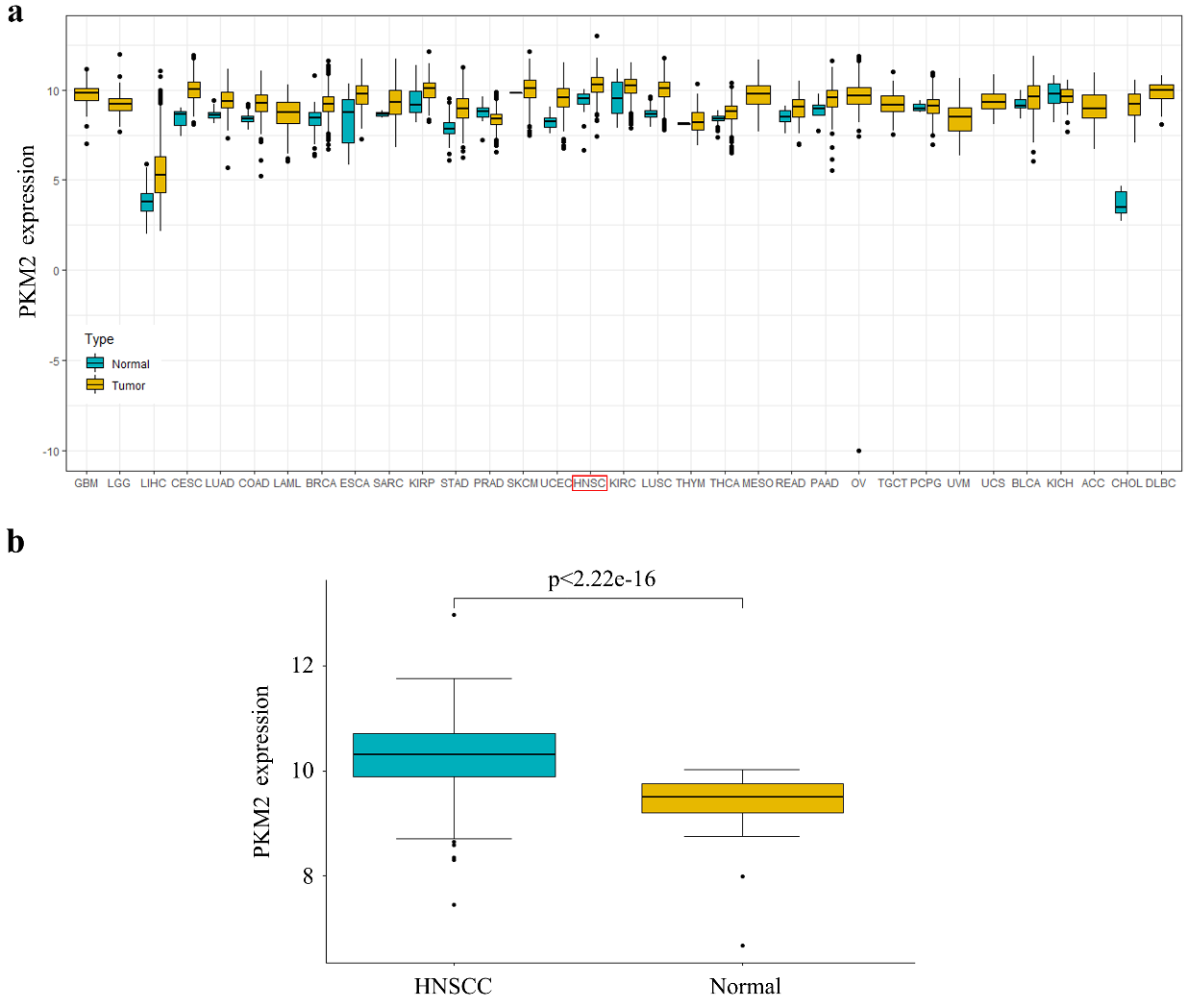

Supplement: Supplementary file 5 — Additional file 5: Supplementary Figure S5. The expression of PKM2 in tumor and normal tissue samples based on TCGA database. (a) Analysis of PKM2 expression in normal and tumor tissues using TCGA cohort with 33 cancer types. (b) Analysis of PKM2 expression in head and neck normal and tumor tissues using TCGA HNSC cohort. [file 13046_2022_2574_MOESM5_ESM.docx]
